# A Novel Triboelectric–Electromagnetic Hybrid Generator with a Multi-Layered Structure for Wind Energy Harvesting and Wind Vector Monitoring

**DOI:** 10.3390/mi16070795

**Published:** 2025-07-08

**Authors:** Jiaqing Niu, Ribin Hu, Ming Li, Luying Zhang, Bei Xu, Yaqi Zhang, Yi Luo, Jiang Ding, Qingshan Duan

**Affiliations:** 1 School of Mechanical Engineering, Guangxi University, Nanning 530004, China; jiaqing@st.gxu.edu.cn (J.N.); pprb8073@163.com (R.H.); liming@binn.cas.cn (M.L.); zly_19117529721@163.com (L.Z.); 2 Beijing Key Laboratory of Micro-Nano Energy and Sensor, Center for High-Entropy Energy and Systems, Beijing Institute of Nanoenergy and Nanosystems, Chinese Academy of Sciences, Beijing 101400, China; 3 School of Light Industry and Food Engineering, Guangxi University, Nanning 530004, China; xubei@st.gxu.edu.cn (B.X.); yaqi9274@st.gxu.edu.cn (Y.Z.); yiluo@st.gxu.edu.cn (Y.L.)

**Keywords:** wind energy harvesting, triboelectric nanogenerators, electromagnetic generator, hybrid mechanism, multi-layered structure, environmental monitoring

## Abstract

High-efficiency wind energy collection and precise wind vector monitoring are crucial for sustainable energy applications, smart agriculture, and environmental management. A novel multi-layered triboelectric–electromagnetic hybrid generator (TEHG) for broadband wind energy collection and wind vector monitoring was built. The TEHG comprises three functional layers corresponding to three modules: a soft-contact rotary triboelectric nanogenerator (S-TEHG), an electromagnetic generator (EMG), and eight flow-induced vibration triboelectric nanogenerators (F-TENGs), which are arranged in a circular array to enable low-wind-speed energy harvesting and multi-directional wind vector monitoring. The TEHG achieves broadband energy harvesting and demonstrates exceptional stability, maintaining a consistent electrical output after 3 h of continuous operation. The EMG charges a 1 mF capacitor to 1.5 V 738 times faster than conventional methods by a boost converter. The TEHG operates for 17.5 s to power a thermohygrometer for 103 s, achieving an average output power of 1.87 W with a power density of 11.2 W/m^3^, demonstrating an exceptional power supply capability. The F-TENGs can accurately determine the wind direction, with a wind speed detection error below 4.5%. This innovative structure leverages the strengths of both EMG and TENG technologies, offering a durable, multifunctional solution for sustainable energy and intelligent environmental sensing.

## 1. Introduction

Wind energy is recognized as a renewable resource that is crucial in addressing global energy challenges [1,2,3]. Its extensive availability and minimal environmental impact position it as a cornerstone for achieving carbon neutrality and advancing sustainable development goals [4,5,6,7]. In addition to its function in generating energy, wind significantly influences atmospheric systems and industrial processes, making precise wind measurement essential for applications such as meteorological forecasting, environmental monitoring, and agricultural planning [8,9,10]. With the growing demand for renewable energy, developing technologies to harness wind energy has become a research focus for sustainability [11,12]. The triboelectric nanogenerator (TENG), developed by Wang’s group, utilizes the combined phenomena of electrostatic induction and contact electrification; it has gained considerable attention as a viable technology for wind energy collection, particularly for powering Internet of Things sensors [13,14,15,16,17]. Furthermore, the TENG-based self-powered sensor can monitor the wind vector, due to its heightened sensitivity to mechanical stimuli [18]. Consequently, these unique features have driven the development of TENG-based systems for both environmental monitoring applications and sustainable energy collection [19].

Currently, there are two main structures of TENGs for harvesting wind energy: flow-induced vibration and rotation [20]. Flow-induced vibration TENGs, such as freestanding flag-type designs, demonstrate an excellent performance in multi-directional energy harvesting, especially under high-altitude conditions [21]. Topology-optimized structures, such as leaf-inspired designs, further enhance their efficiency by reducing electrostatic adsorption and lowering the cut-in wind speed [22]. Xie et al. first combined rotational TENGs with traditional wind power technology to transform wind energy into electricity, highlighting the TENG’s potential as a wind power technology [23]. Meanwhile, rotational TENGs exhibit a superior electrical output, making them ideal for powering small-scale electronics; however, challenges such as material wear and reduced performances at low wind speeds remain significant barriers to their broader application [24]. Compared to rotational TENGs, flow-induced vibration TENGs have a lower start-up wind speed and are more sensitive to wind. The output of flow-induced vibration TENGs exhibits high-frequency characteristics under wind excitation from specific directions, making them suitable for wind direction and speed monitoring in natural environments [25]. Xu et al. introduced a flow-induced vibration TENG based on aeroelastic flutter under high-humidity environments, which functions as a dynamic wind speed sensor by analyzing the flutter frequency, showing its potential in wireless environmental monitoring systems [26].

Although significant progress has been made in TENG-based self-powered sensing and wind energy collection, single-mechanism energy harvesters often experience substantial energy losses, resulting in a low conversion efficiency [27]. To solve this limitation, piezoelectric generators and electromagnetic generators (EMGs) have been integrated with TENGs to enhance electrical output and provide additional functionalities, such as sensing [28,29]. For instance, Bai et al. introduced a hybrid wind energy collector integrating electromagnetic, piezoelectric, and triboelectric mechanisms with a snap-through bistable design, where the rapid switching deformation driven by magnetic excitation enables the synergistic operation of the three energy conversion mechanisms, enabling self-powered wind speed monitoring in real-time and enhancing the electrical output in extreme environments [30]. Most existing systems prioritize energy conversion efficiency, often overlooking wind vector monitoring, which is crucial for weather forecasting and human safety. Therefore, it is essential to design a hybrid system that combines high energy efficiency, multi-directional wind vector detection, and operational stability over extended periods.

Compared to hybrid harvesters with limited functionality, this paper introduces a novel triboelectric–electromagnetic hybrid generator (TEHG) featuring a multi-layered design, achieving the dual functionality of broadband wind energy harvesting and real-time wind vector monitoring. The TEHG has three layers corresponding to the three modules: a soft-contact rotary triboelectric nanogenerator (S-TEHG), an EMG, and eight flow-induced vibration triboelectric nanogenerators (F-TENGs). By significantly broadening the operational wind spectrum through the low cut-in wind speed of the F-TENG, and enabling real-time wind direction/speed monitoring via a circular F-TENG array, the TEHG achieves broadband energy harvesting. This paper comprehensively evaluates the electrical output of the TEHG under various wind speeds, focusing on broadband wind energy harvesting, accurate wind vector monitoring, and excellent electrical output stability. The soft-contact mode endows the S-TENG and EMG to achieve a low cut-in wind speed (3.3 m·s^−1^) and maintain a stable electrical output after 3 h (16,200 cycles) of continuous operation. Additionally, a platform is designed with LabVIEW 2020 for real-time wind vector monitoring. Compared to conventional power supply methods, integrating a boost converter into the hybrid power management circuit achieves a 738-fold increase in the charging speed, significantly enhancing the energy storage efficiency. This enables the TEHG to continuously power a thermohygrometer for 103 s at 6.0 m·s^−1^. Overall, this work proposes a multi-layered structure with a hybrid energy harvesting mechanism, highlighting its significant potential for both wind monitoring and energy collection.

## 2. Materials and Methods

### 2.1. The Fabrication of the TEHG

The TEHG integrates three main components: the S-TENG, F-TENGs, and EMG. The S-TENG comprises a rotor, stator, and wind cups, all fabricated from resin material (PLA) using 3D printing. Six wind cups (diameter: 80 mm, arm length: 150 mm, thickness: 1.5 mm) are mounted on the top of the central shaft. The rotor (diameter: 107 mm, height: 105 mm, thickness: 1.5 mm) and stator (diameter: 130 mm, height: 127 mm, thickness: 1.5 mm) are connected to the central shaft (diameter: 10 mm, length: 200 mm) via ceramic bearings positioned on the lower and upper surfaces of the stator (inner diameter: 10 mm, thickness: 9 mm, outer diameter: 30 mm). Six arched FEP films (length: 100 mm, width: 90 mm, thickness: 0.03 mm) are evenly affixed to the outer surface of the rotor. Twelve Cu electrodes (length: 49 mm, width: 100 mm, thickness: 0.06 mm, spacing: 2 mm) are fixed on the inside of the stator.

The EMG primarily consists of two coils (diameter: 30 mm, height: 10 mm, wire diameter: 0.4 mm) and eight magnets (thickness: 5 mm, diameter: 30 mm).

The overall structure of the F-TENGs (diameter: 160 mm, height: 70 mm) is fabricated from resin material (PLA) using 3D printing. The top and bottom plates have a diameter of 160 mm and a thickness of 5 mm. The F-TENGs are composed of 16 rectangular air deflectors (length: 70 mm, width: 60 mm, thickness: 2 mm). FEP films (thickness: 0.015 mm, width: 50 mm, length: 65 mm) are fixed in the center of the rectangular air deflectors using 5 mm thick sponge tape. Copper foil (thickness: 0.06 mm, width: 60 mm, length: 70 mm) is applied to the inside of the rectangular air deflectors. Eight F-TENGs are positioned in a circular arrangement on the base plate. The plate spacing of the F-TENG is 10 mm.

### 2.2. Electrical Measurements and Simulations

The blower (HF-315P, Hongguan, Shanghai, China) with a variable frequency drive (EV4300, Maifu Electric, Yancheng, China) is used to generate the required wind force for the experiments. The *V_OC_* and *I_SC_* are obtained with a programmable electrometer (Keithley 6514, Tektronix, Beaverton, OR, USA), while the voltage simulation is conducted by COMSOL Multiphysics 6.0 software. For multi-channel measurements and demonstrations, the voltage output is recorded by an ADC (DAQ 122, 8-channel voltage measurement module, ±10 V, LOCKZHINER Electronic, Fuzhou, China). The boost converter used in the EMG module is a BL8531-3.3V (AETHER, Changzhou, China).

## 3. Results and Discussion

### 3.1. Composition and Working Principle of TEHG

**Figure 1:**
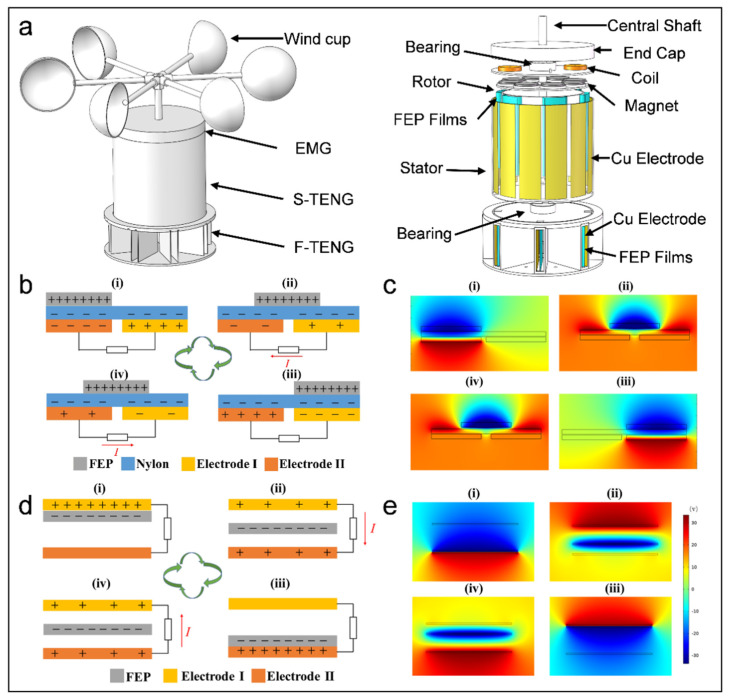
Composition and working principle of TEHG. (**a**) Composition of TEHG. (**b**) Working principle and (**c**) voltage distribution model of S-TENG. (**d**) Working principle and (**e**) voltage distribution model of F-TENG.

The TEHG features a three-layer structure (Figure 1a), with each layer corresponding to the three modules: an EMG, an S-TEHG, and F-TENGs. The S-TENG primarily comprises wind cups, a rotor, and a stator, designed with an inner rotor and an outer stator structure. The wind cups are driven by wind and attached to the rotor via bearings and a central shaft. Six arches made of FEP film are evenly mounted on one side of the outer rotor wall, making contact with twelve Cu electrodes affixed to the inner stator wall. By designing the FEP film into an arched structure to achieve a soft-contact mode, its good elasticity can effectively reduce friction and minimize wear [27,31]. There are eight F-TENGs in a freestanding mode, and each contains two rectangular air deflectors in the F-TENG. Cu electrodes are glued to the inner walls of these deflectors, and the FEP film is bonded to them using sponge tape, leaving a gap for the wind to flow and thereby inducing vibration. The eight F-TENGs are arranged in a circular array and fixed in eight directions on the base plate to enable wind vector monitoring. The EMG consists of magnets and coils, with the magnets on the rotor and the series-connected coils positioned on the stator. Figures S1 and S2 depict the physical diagrams of the TEHG components.

Figure 1b depicts the working principle of the S-TENG. The Cu electrode on the left is designated Electrode I, and Electrode II is the other. The process of electricity generation over an entire cycle is as follows: (i) Initially, the Nylon film above Electrode I and the FEP are in full contact (Figure 1(bi)). (ii) Under the influence of wind, the FEP film rotates with the rotor and partially contacts the Nylon film above Electrode II. Due to the voltage disparity between the electrodes, electron migration is triggered, establishing a current across the external path from Electrode II to Electrode I (Figure 1(bii)). (iii) The FEP film fully covers the Nylon film above Electrode II, prompting a complete electron transfer between Electrode II and Electrode I (Figure 1(biii)). (iv) Finally, the FEP film persists in its movement and makes contact with the subsequent Nylon film above Electrode I. This movement triggers electron migration, establishing a current across the external path from Electrode I to Electrode II, thus a power generation cycle is finished (Figure 1(biv)). The voltage distribution model over a whole cycle is simulated using COMSOL, providing deeper insight (Figure 1c). The specific simulation process is mentioned in Supplementary Note S1.

Figure 1d depicts the working principle of the F-TENG. The upper and lower Cu electrodes are designated as Electrode I and Electrode II, respectively. The process of the electricity generation process over an entire cycle is as follows: (i) Electrode I and the FEP film are in full contact. Both the FEP film and Electrode I acquire charges of equal magnitude but an opposite polarity due to electrostatic induction (Figure 1(di)). (ii) Under the influence of wind, the FEP film swings away from Electrode I and approaches Electrode II. This movement triggers electron migration, establishing a current across the external path from Electrode I to Electrode II (Figure 1(dii)). (iii) The FEP film and Electrode II are in full contact, acquiring charges of equal magnitude but an opposite polarity (Figure 1(diii)). (iv) Finally, as the FEP film oscillates back toward Electrode I, electrons migrate to establish a current across the external path from Electrode II to Electrode I, thereby finishing an electricity generation cycle (Figure 1(div)). Figure 1e shows the voltage distribution model over a whole cycle simulated using COMSOL.

Once the ambient wind speed is below the starting threshold of the wind cups, the flexible film of the F-TENG harvests wind energy by fluttering in the low-speed airflow, significantly extending the operating wind speed range of the device. Once the ambient wind speed reaches the starting threshold of the wind cups, all modules of the TEHG operate synergistically to enhance the energy harvesting efficiency. Owing to its circumferential array configuration, the F-TENG establishes a spatial mapping relationship between its output electrical signals and the wind field vectors. This enables real-time wind direction derivation, while the inherent frequency response characteristics of these signals form the basis for robust ambient wind speed monitoring. The TEHG leverages synergistic interactions among modular components and the spatially optimized structural design. This approach facilitates the collection of broadband wind energy while simultaneously incorporating the ability to monitor environmental wind vectors in real-time.

### 3.2. Optimization of Output Characteristics for TEHG

#### 3.2.1. Output Characteristics of S-TENG

**Figure 2:**
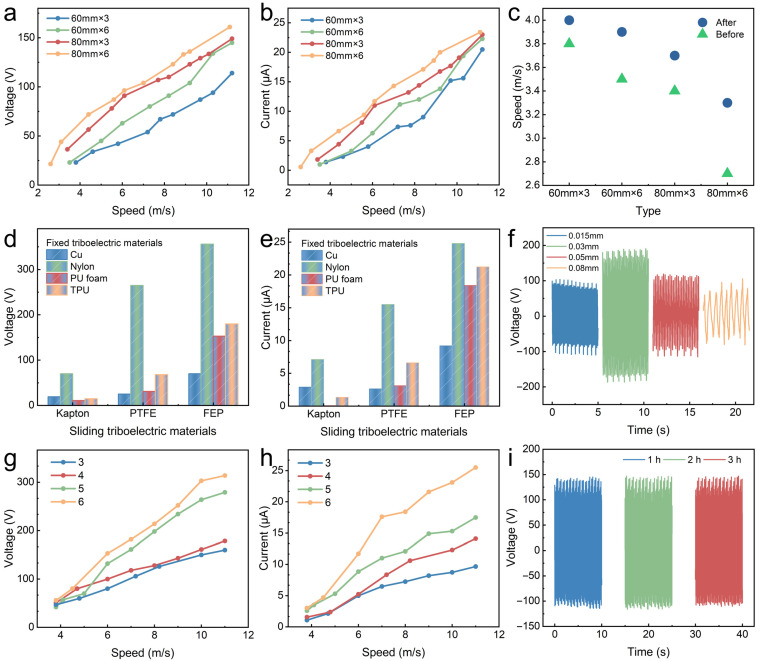
Optimization of S-TENG output. (**a**–**c**) Electrical output and cut-in wind speed using different wind cup structures. Electrical output of S-TENG: (**d**,**e**) with different triboelectric material combinations (8.0 m·s^−1^), (**f**) with different thicknesses of FEP (6.0 m·s^−1^), (**g**,**h**) and with different numbers of electrode pairs under various wind speeds. (**i**) Stability test.

To examine the electrical output of the TEHG in various wind conditions, a blower with a frequency converter was employed to generate wind. Winds of varying speeds were generated by adjusting the frequency of the frequency converter. All experiments were conducted in a controlled laboratory environment (temperature: 27 ± 2 °C, relative humidity: 50 ± 5%) to isolate the wind speed as the primary variable. Numerous studies have shown that the materials and structure of the S-TENG significantly influence its electrical output [32,33,34,35]. The effects of the wind cup size and number, triboelectric material, film thickness, and number of electrode pairs on the outputs of the S-TENG were comprehensively examined. All experiments in this work were designed as single-factor tests with controlled variables. The cut-in wind speed of the structure was significantly influenced by the type of wind cups, primarily due to differences in their mass and wind-capturing efficiency. With other variables held constant, different quantities and diameters of wind cups were selected to compare their output performance. With six wind cups measuring 80 mm in diameter, the S-TENG demonstrated a superior output performance, significantly surpassing other wind cup configurations (Figure 2a,b). After attaching magnets to the rotor, the increased rotor mass led to a higher cut-in wind speed (3.3 m·s^−1^), meeting the requirements for low-wind-speed operations (Figure 2c). Therefore, a set of six wind cups with a diameter of 80 mm was chosen as the wind-facing mechanism for the S-TENG. As indicated in Table 1, the cut-in wind speed of the wind energy collector presented in this study was compared with that reported in the representative literature from recent years.

Since different materials have different abilities in gaining and losing electrons and retaining a charge, the outputs of the S-TENG are significantly affected by different triboelectric pairs [38]. The positive triboelectric materials (fixed triboelectric materials) include Cu, Nylon, PU foam, and TPU, while the negative triboelectric materials (sliding triboelectric materials) include Kapton, PTFE, and FEP. The S-TENG exhibits an optimal electrical performance when the triboelectric materials are Nylon and FEP (Figure 2d,e).

Furthermore, with the FEP film thickness increasing, the open-circuit voltage (*V_OC_*) and short-circuit current (*I_SC_*) initially first rise and subsequently decline, with an optimal thickness of 0.03 mm (Figure 2f and Figure S3). This can be attributed to the observation that under the same wind speed conditions, thinner FEP films demonstrated increased deformation, which increases the contact area with the Cu electrode and thereby improves the electrical output. However, excessively thin FEP films may collapse in the middle section due to insufficient structural support, leading to reduced contact with the Cu electrode and a diminished electrical output. Conversely, thicker FEP films exhibit reduced elasticity, limiting the contact area and lowering the electrical performance. Additionally, the increased friction between the thicker films and the Cu electrode raises the required cut-in wind speed. Based on these considerations, an FEP film thickness of 0.03 mm was identified to be the most suitable option for the S-TENG. Generally, increasing the number of electrode pairs significantly improves the rate of the charge transfer between the Cu electrodes, resulting in a higher *I_SC_* [39]. The S-TENG with six pairs of Cu electrodes exhibited an optimal electrical output, owing to the increased contact area of the triboelectric materials (Figure 2g,h). However, when excessive electrode pairs are present, adjacent FEP films make contact with each other, leading to the coverage of neighboring electrodes. Therefore, freestanding layers with six pairs of Cu electrodes were determined to be the most effective configuration. Owing to the soft-contact operation mode of the S-TENG, the peak output voltage maintained 99.3% of its initial value after 3 h (16,200 cycles) at 8.0 m·s^−1^ (Figure 2i), demonstrating its stability during a long-term operation.

#### 3.2.2. Electrical Characteristics of F-TENG

A systematic investigation was conducted to assess the impact of key parameters, including the triboelectric materials, film dimensions (area and thickness), and plate spacing, aiming to optimize the performance of the F-TENG and enable multi-directional wind vector monitoring.

**Figure 3:**
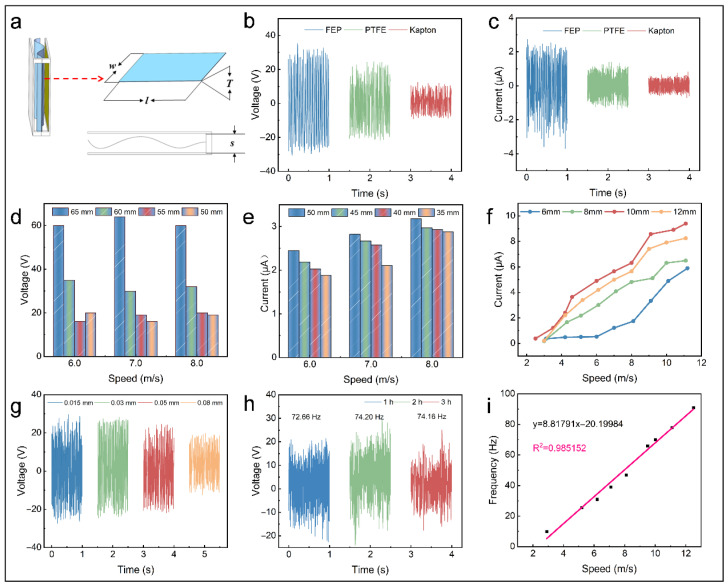
Optimization of F-TENG output. (**a**) Schematic image of F-TENG structure. Electrical performance of F-TENG: (**b**,**c**) with different triboelectric materials (7.0 m·s^−1^), (**d**,**e**) with different FEP areas under various wind speeds, (**f**) with varying plate spacings, (**g**) and with different thicknesses of FEP (6.0 m·s^−1^). (**h**) Stability test. (**i**) Fitting curve of electrical signal frequency versus wind speed.

Due to its low friction resistance and sensitivity to mechanical stimuli, the F-TENG can start operating under extremely low wind speeds [7]. However, because the wind speed of the experimental blower is highly unstable below 2.8 m·s^−1^, this is considered the lower limit of the test wind speed. Figure 3a illustrates the key parameters of the F-TENG that require optimization. The initial structural dimensions were set as follows: length (*l*) = 65 mm, thickness (*T*) = 0.05 mm, width (*w*) = 50 mm, and plate spacing (*s*) = 10 mm. The electrical outputs of the F-TENG with various negative triboelectric materials were tested at 7.0 m·s^−1^ (Figure 3b,c). It was evident that the FEP film exhibited the best electrical performance, achieving a peak *V_OC_* of approximately 60 V, followed by PTFE, while Kapton showed the lowest performance. As the film area increases, the electrical output of the F-TENG demonstrates an increasing trend. This facilitates charge accumulation on the triboelectric layers, owing to the increased contact area between the materials, thereby enhancing the output (Figure 3d,e). The plate spacing influences the flapping frequency of the film and charge transfer rate, resulting in the varying performance of the F-TENG. The F-TENG achieves an optimal electrical output with a plate spacing of 10 mm. This phenomenon occurs because reduced spacing induces incomplete contact–separation cycles due to interlayer adhesion, which lowers the charge transfer efficiency, while excessive spacing prolongs contact intervals and thus significantly diminishes the contact frequency (Figure 3f). The *V_OC_* of the F-TENG with varying FEP thicknesses was measured under the wind speed of 6.0 m·s^−1^. The results showed that thinner films produced a higher triboelectric output, with the best performance achieved at an FEP thickness of 0.015 mm (Figure 3g). Using Hooke’s Law, Zhao et al. proposed that reducing the film thickness increases the force applied to the film, thereby improving the contact between the triboelectric materials [40,41]. Therefore, the selected FEP film dimensions were 65 mm (*l*) × 50 mm (*w*) × 0.015 mm (*T*), with a plate spacing of 10 mm.

The wind speed is a crucial factor that greatly impacts the accuracy of weather forecasts. This study evaluated the capability of the F-TENG for real-time wind speed monitoring. In light of the working mechanism of the F-TENG, an electrical signal arises from the periodic contact between the film and electrode under wind excitation. However, the F-TENG’s voltage is highly unstable and susceptible to the temperature, humidity, and other environmental factors, making it difficult to establish a direct correlation with wind speed. Therefore, it is generally unsuitable as a reliable signal source for wind speed monitoring. In contrast, the frequency of electrical signals is strongly correlated with the film flapping frequency, which in turn correlates strongly with the wind speed. Therefore, the frequency of electrical signals can serve as a reliable signal source for wind speed sensing [22,41,42]. Figure 3h illustrates the F-TENG’s voltage and the frequency of the electrical signal at 6.0 m·s^−1^. It operates stably and continuously for 3 h, indicating its potential as an essential parameter for monitoring the wind vector. The investigation of the connection between the wind speed and the frequency of electrical signals demonstrates a nearly linear correlation between the two variables (Figure 3i).

#### 3.2.3. Output Characteristics of EMG

The EMG is a crucial component of the TEHG, which is characterized by a high output current and a low output voltage [27,30]. To improve the efficiency of the multi-directional wind energy collection, this study systematically explored the electrical output of the EMG using varying wind cup types, magnet sizes, and coil–magnet combinations.

**Figure 4:**
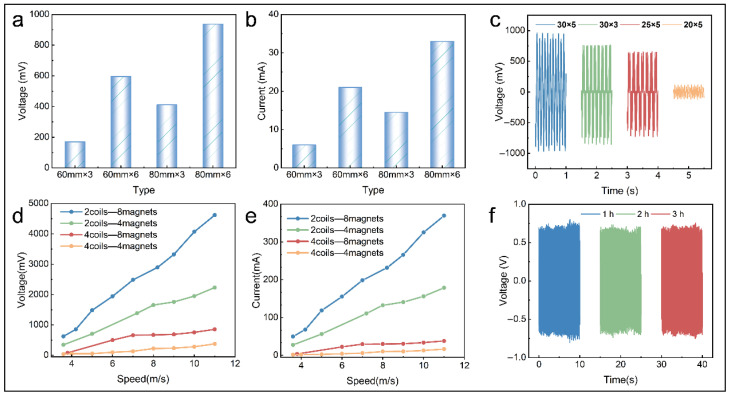
Optimization of EMG output. Electrical output of EMG: (**a**,**b**) with different wind cup types (6.0 m·s^−1^), (**c**) with different magnet sizes (6.0 m·s^−1^), and (**d**,**e**) with varying coil–magnet combinations. (**f**) Stability test.

The electrical output of the EMG was proportional to both the diameter and number of the wind cups (Figure 4a,b). The EMG exhibited an optimal electrical output using a wind-facing mechanism consisting of six wind cups, each with a diameter of 80 mm. Notably, the cut-in wind speed of the mechanism is influenced by the mass (number and size) of the magnets. Magnets of different dimensions exhibit distinct magnetic moment densities. According to Faraday’s Law of Electromagnetic Induction, increasing the effective volume of magnets enhances the air-gap flux density. This enhancement consequently elevates the rate of the magnetic flux variation through EMG coils, resulting in a proportional improvement of the electrical output performance [43]. The EMG, with magnets 30 mm in diameter and 5 mm in thickness, demonstrated the optimal electrical output (Figure 4c).

After selecting the wind cup type and magnet size, the electrical outputs of the EMG were examined for various combinations of magnets and coils. The optimal combination was selected by evaluating the electrical output of different coil–magnet combinations. The combination of eight magnets and two coils was found to be the best, and the peak voltage reached approximately 4.8 V at 11.0 m·s^−1^, possibly because alternating polarities of adjacent magnets induce high-frequency magnetic field reversals during rotation. With two symmetrically positioned coils angularly spaced 180° apart, their induced voltages maintain a precise phase synchronization, enabling the voltage superposition through the series connection. Conversely, when four coils are arranged at 90° intervals, phase differences in magnetic field variations between adjacent coils cause a partial voltage cancelation (Figure 4d,e). The output of the EMG is positively correlated with the rate of the magnetic flux variation, which is relatively stable at a fixed wind speed, so the voltage showed little decrease after 3 h of operation at 5.0 m·s^−1^ (Figure 4f), illustrating the durability and stability of the EMG during a long-term operation.

### 3.3. The Electrical Output Characteristics of the TEHG

**Figure 5:**
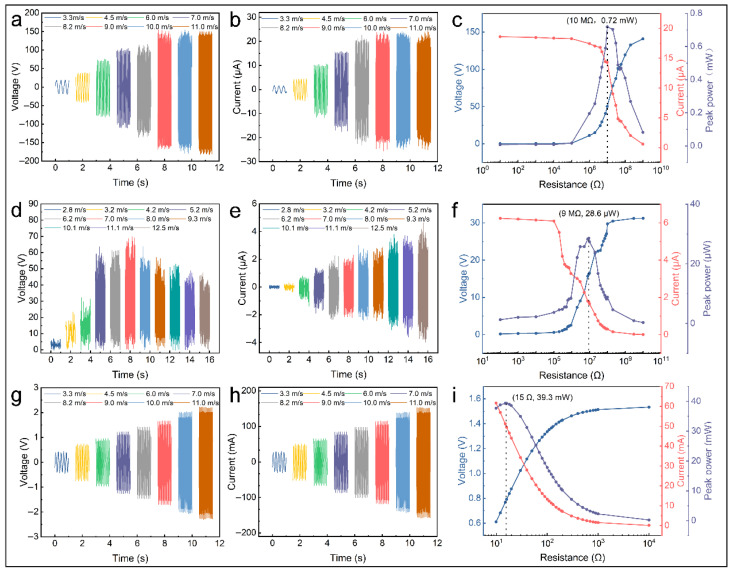
Electrical performances of TEHG. Electrical performance of S-TENG: (**a**) *V_OC_* and (**b**) *I_SC_* at varied wind speeds and (**c**) external load power (8.0 m·s^−1^). Electrical performance of EMG: (**d**) *V_OC_* and (**e**) *I_SC_* at varied wind speeds and (**f**) external load power (8.0 m·s^−1^). Electrical performance of F-TENG: (**g**) *V_OC_* and (**h**) *I_SC_* at varied wind speeds and (**i**) external load power (8.0 m·s^−1^).

After optimizing the individual modules of the TEHG, this section systematically presents the electrical performance of each module. The evaluation was conducted at various wind conditions to analyze the electrical performance of the S-TENG module (Figure 5a,b). The peak *V_OC_* and peak *I_SC_* were approximately 40 V and 3.6 μ A at 3.3 m·s^−1^, respectively, and they tend to stabilize with the wind speed increasing. The peak *V_OC_* reached approximately 314 V, and the peak ISC reached approximately 43 μA at 11.0 m·s^−1^. This trend is partly due to the soft-contact working mode and partly because the arched FEP film lacks support during the start-up phase, resulting in insufficient contact with the triboelectric material and fewer triboelectric charges. The rotational speed accelerates as the wind speed increases, resulting in more contact between the triboelectric materials. This enhances the generation and transfer of triboelectric charges, stabilizing once saturation is reached. Experimental measurements of the electrical output at various loads revealed a significant nonlinear relationship between the output power and load resistance at 8.0 m·s^−1^ (Figure 5c). Upon adjusting the load resistance to 10 MΩ, the S-TENG achieves optimal impedance matching. The peak power is 0.72 mW, with the corresponding current and voltage of 14.3 μA and 50.3 V, respectively.

For the F-TENG, high-frequency flapping causes insufficient contact between the triboelectric materials, reducing the transferred charge, so the voltage first rises and then declines with the rising wind speed. It reaches its peak *V_OC_* of 70 V at 7.0 m·s^−1^ (Figure 5d). Meanwhile, the high-frequency flapping of the film reduces the time interval, thereby enhancing the rate of the charge transfer [33,43,44]. Therefore, the current increases as the wind speed increases, reaching its peak *I_SC_* of 8.5 μA at 11.1 m·s^−1^ (Figure 5e). Upon adjusting the load resistance to approximately 9 MΩ, the F-TENG achieves optimal impedance matching. The peak power is 28.6 μW, with the corresponding current and voltage at this peak power being 1.76 μA and 16.2 V, respectively (Figure 5f).

For the EMG, Faraday’s law indicates that its electrical output is related to changes in the magnetic flux [43]. These changes are determined by the differential rotational speed between the magnets and coils, which increases with the wind speed. The peak *V_OC_* reaches approximately 4.5 V at 11.0 m·s^−1^ (Figure 5g,h). Upon adjusting the load resistance to approximately 15 Ω, the EMG achieves optimal impedance matching. The peak power is 39.3 mW, and the corresponding voltage and current are 0.77 V and 51.22 mA, respectively, indicating a superior electrical output performance (Figure 5i). As shown in Table 2, the relevant information is listed to highlight the output performance advantages of the TEHG.

### 3.4. Demonstrations

**Figure 6:**
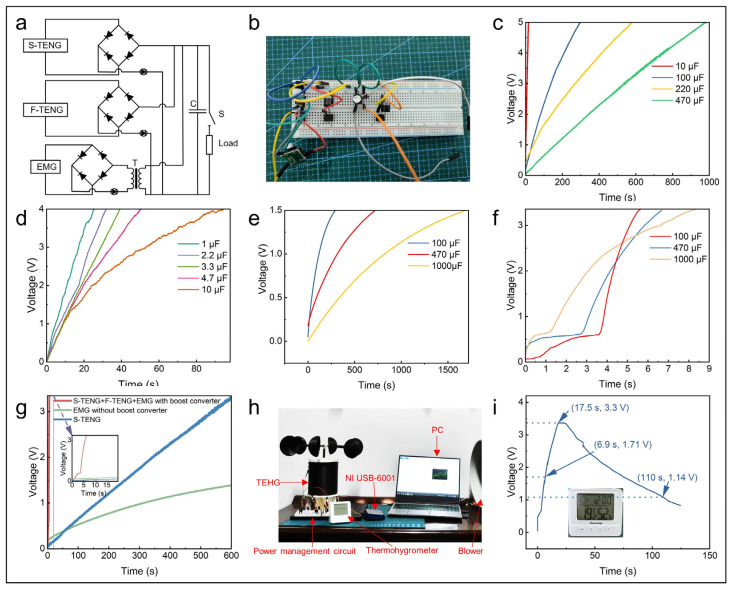
A demonstration of the TEHG as a power supply. (**a**,**b**) Schematic and physical images of the circuit. Charging different capacitors by the (**c**) S-TENG, (**d**) F-TENG, and (**e**) EMG without a boost converter, (**f**) the EMG with a boost converter, (**g**) and the TEHG. (**h**) A physical picture of the TEHG and (**i**) the voltage of the storage capacitor while charging the thermohygrometer.

The performance of the TEHG is rectified through the power management circuit and is stored in a capacitor, which subsequently powers the electronic devices (Figure 6a,b). The S-TENG effectively charged a 10 μF capacitor to 5 V within 15 s at 6.0 m·s^−1^ (Figure 6c), while the F-TENG required 91 s to charge to 4 V (Figure 6d). Due to the output voltage of the EMG being below 1 V, the capacitor charging rate was relatively slow (Figure 6e). To address this, a boost converter was employed after the diode rectification to achieve additional low-frequency voltage boosting. With a boost converter, the EMG charged a 1000 μF capacitor to 3.3 V in 8.5 s (Figure 6f) and to 1.5 V 738 times faster than without a boost converter. The hybrid power supply further demonstrated a superior performance by charging a 470 μF capacitor to 3.3 V within just 6.3 s (Figure 6g). With the integration of the boost converter, the TEHG successfully delivered a stable 3.3 V output, making it suitable for powering small electronic devices. The TEHG charged six parallel 1000 µF capacitors to 3.3 V in 17.5 s at 6.0 m·s^−1^. Upon reaching 1.71 V, it powered a thermohygrometer. After the charging process ceased, it could continue to operate for ~93 s (Figure 6h,i, and Movie S1). The data on the stored energy, output average power, and power density throughout the charging process are presented in Table 3. In conclusion, the above tests demonstrated the potential of the TEHG as a power supply.

**Figure 7:**
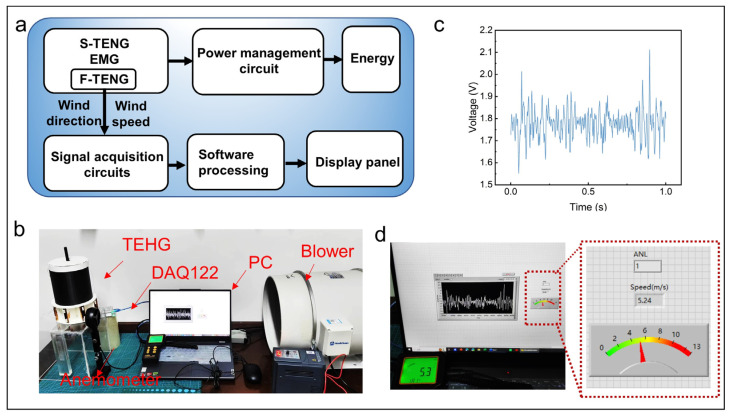
A demonstration of the wind vector monitoring by the F-TENGs. (**a**) The working principle flowchart of the TEHG. (**b**) The experimental platform for real-time wind vector monitoring. (**c**) Electrical signals of the channel facing the wind direction (5.3 m·s^−1^). (**d**) The wind vector display panel.

Figure 7a shows the working principle flowchart of the TEHG. The TEHG serves dual functions: it continuously powers small electronic devices and enables wind vector monitoring in real-time through the electrical signal analysis from each channel of F-TENGs. The F-TENGs feature an octant-based spatial distribution design, with channels 1 to 8 arranged clockwise to correspond with eight cardinal directions. The channel of F-TENGs that faces the wind generates an electrical signal when exposed to wind, while the other channels do not due to the absence of wind. Therefore, the electrical signals from the various channels indicate different wind directions, with channel numbers 1 to 8 corresponding to specific directions. To validate the performance of the TEHG and monitor environmental wind parameters, an experimental platform is constructed (Figure 7b). The platform integrates data acquisition, signal processing, and real-time monitoring functionality. Figure 7c shows the raw electrical signals for channel 1 (the channel facing the wind direction) of the eight-channel system. A platform for real-time wind direction and speed monitoring was designed by LabVIEW 2020. The electrical signals from each channel of the F-TENGs are collected by an 8-channel voltage measurement module. After the signals are processed and analyzed by LabVIEW 2020, the wind direction can be identified according to the corresponding channels, while the wind speed is derived using a linear equation that relates the wind speed to the frequency of the electrical signal, enabling the accurate monitoring of the wind vector in real-time. Due to the voltage from each channel of the F-TENGs exceeding the range of the eight-channel voltage measurement module, a 20 MΩ resistor was connected in parallel to each channel to measure the voltage. Figure 7d and Movie S2 depict the processed wind direction and speed. Experiments show that F-TENGs can accurately monitor the wind vector for wind speeds between 2.8 and 12.5 m·s^−1^, with the wind speed measurement error remaining within 4.5% (Table S1). We compared the wind vector detection capabilities of the F-TENGS with previous representative works, demonstrating the advantages of the F-TENGS in wind vector monitoring (Table 4).

## 4. Conclusions

This paper proposes a novel TEHG with a multi-layered structure for the precise monitoring of the wind vector and effective wind energy harvesting, addressing limitations of single-mechanism devices. The TEHG has three layers corresponding to the three modules: an EMG, an S-TEHG, and F-TENGs arranged in a circular array. Through innovative design and parameter optimization, the TEHG achieves broadband energy harvesting, with the EMG and S-TENG achieving a low cut-in wind speed (3.3 m·s^−1^). The peak power outputs of the TEHG are 0.72 mW (S-TENG), 28.6 μW (F-TENG), and 39.3 mW (EMG) at 7.0 m·s^−1^, demonstrating excellent energy harvesting efficiency. By utilizing a multi-layer structure design combined with a hybrid energy mechanism, the TEHG demonstrates excellent durability and stability, maintaining a stable electrical output after a prolonged operation. Additionally, by introducing a boost converter into the power management circuit, the TEHG can charge six parallel 1000 µF capacitors to 3.3 V within 17.5 s, achieving an average output power of 1.87 W with a power density of 11.2 W/m^3^, enabling a thermohygrometer to operate continuously for 103 s (6.0 m·s^−1^). The circular array configuration of F-TENGs enables precise multi-directional wind vector detection with an error rate of less than 4.5% (2.8~12.5 m·s^−1^). This paper provides valuable guidance for deploying distributed sensors and efficiently harvesting wind energy using renewable resources, showcasing its significant potential in smart agriculture and environmental monitoring. Future work will focus on the following aspects to address this study’s limitations: (1) Optimizing the wind-facing structure to reduce the device’s cut-in wind speed. (2) Systematically studying how environmental factors like temperature and humidity affect the TEHG’s output characteristics. (3) Conducting operation tests in extreme weather conditions like rain and snow to enhance the TEHG’s environmental adaptability and practical value.

## Figures and Tables

**Table 1:** Comparison of cut-in wind speed between this work and previous representative works.

Reference	Mechanism	Cut-In Wind Speed	Diameter	Number
[32]	TENG	3.3 m/s	50 mm	6
[36]	TENG	5 m/s	50 mm	6
[37]	TENG + PEG + EMG	3 m/s	35 mm	3
[1]	TENG + EMG	4 m/s	-	6
[30]	TENG + PEG + EMG	3 m/s	60 mm	6
[28]	TENG + EMG	4.3 m/s	-	3
This work	TENG + EMG	3.3 m/s	80 mm	6

**Table 2:** Comparison of electrical output performance between this study and previous representative works.

Reference	Mechanism	Wind Speed	Power
[30]	TENG + PEG + EMG	5 m/s	0.65 mW
[32]	TENG	4 m/s	2.81 mW
[45]	PEG + EMG	6.5 m/s	19.24 mW
[46]	TENG + EMG	9 m/s	18.96 mW
[47]	TENG + EMG	4 m/s	0.32 mW
[48]	TENG + PEG + EMG	3 m/s	11.83 mW
This work	TENG + EMG	8 m/s	40.05 mW

**Table 3:** Performance data during the charging process.

Energy	Power	Power Density
32.67 mJ	1.87 W	11.2 W/m^3^

**Table 4:** Comparison of wind vector monitoring capabilities between this work and previous representative works.

Reference	Wind Speed Range	Wind Direction
[25]	3.77~11.91	×
[26]	3.1~10.8	×
[49]	1.7~6.7	√
[50]	3.0~7.5	√
[51]	2.9~4.5	×
[52]	2.0~12.0	×
[53]	2.0~12.0	×
[54]	2.7~8	√
[33]	4.5~12.5	×
[55]	3~5	√
[56]	1.6~9.4	×
This work	2.8~12.5	√

## Data Availability

The original contributions presented in the study are included in the article; further inquiries can be directed at the corresponding author.

## Supplementary Materials

The following supporting information can be downloaded at: https://www.mdpi.com/article/10.3390/mi16070795/s1, Figure S1. Physical pictures of the TEHG components. (a) The TEHG. (b–e) The rotor, stator, and wind cup of the S-TENG. (f,g) The magnet and coil of the EMG. (h) The F-TENG. Figure S2. Arched FEP film. Note S1: COMSOL simulation process of S-TENG. Figure S3. The ISC of the S-TENG with different thicknesses of FEP under the wind speed of 6.0 m·s^−1^. Table S1. Measured data of F-TENGs, including different channels, actual wind speed, frequency, calculated wind speed, and error. Movie S1: Self-power. Movie S2. Monitoring real-time wind.
