# Supplementary material for: A Novel Triboelectric–Electromagnetic Hybrid Generator with a Multi-Layered Structure for Wind Energy Harvesting and Wind Vector Monitoring
Source: Micromachines (Basel). 2025 Jul 8;16(7):795. doi: 10.3390/mi16070795 (PMC12299051; doi:10.3390/mi16070795)
Supplement: Supplementary file 1 [file micromachines-16-00795-s001.zip › micromachines-3739451-supplementary.pdf]

Supplementary Information

A Novel Triboelectric–Electromagnetic Hybrid Generator with a Multi-Layered Structure for Wind Energy Harvesting and Wind Vector Monitoring

Jiaqing Niu [1], Ribin Hu [1], Ming Li [1,2], Luying Zhang [1], Bei Xu [3], Yaqi Zhang [3], Yi Luo [3], Jiang Ding [1,*] and Qingshan Duan [3,*]

1 School of Mechanical Engineering, Guangxi University, Nanning 530004, China; jiaqing@st.gxu.edu.cn (J.N.); pprb8073@163.com (R.H.); liming@binn.cas.cn (M.L.); zly_19117529721@163.com (L.Z.)
2 Beijing Key Laboratory of Micro-Nano Energy and Sensor, Center for High-Entropy Energy and Systems, Beijing Institute of Nanoenergy and Nanosystems, Chinese Academy of Sciences, Beijing 101400, China
3 School of Light Industry and Food Engineering, Guangxi University, Nanning 530004, China; xubei@st.gxu.edu.cn (B.X.); yaqi9274@st.gxu.edu.cn (Y.Z.); yiluo@st.gxu.edu.cn (Y.L.)
* Correspondence: jding@gxu.edu.cn (J.D.); qs_duan@gxu.edu.cn (Q.D.)

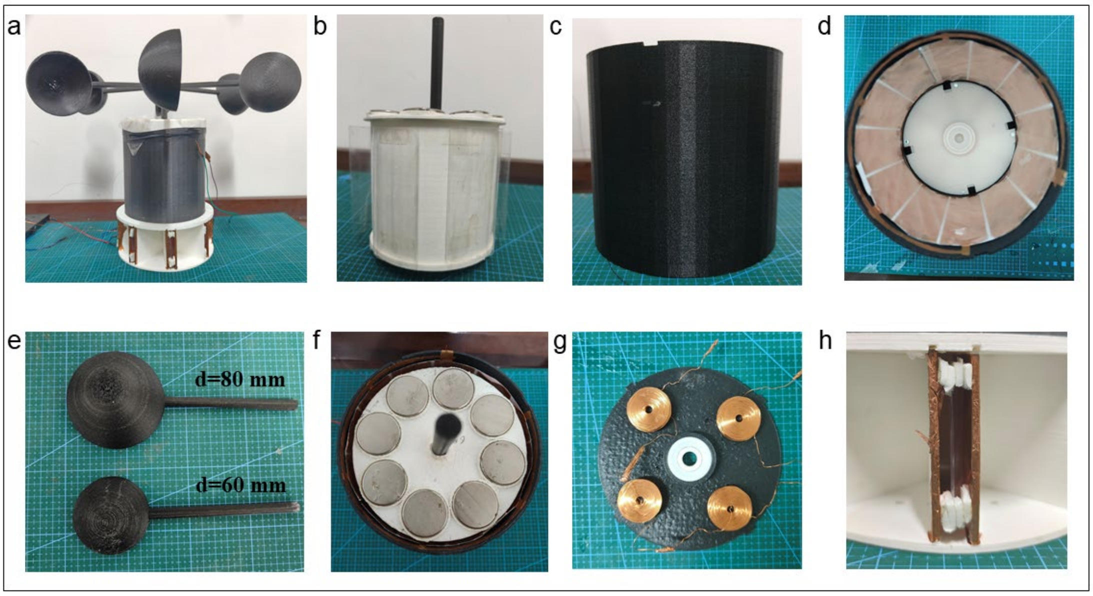

Figure S1. Physical pictures of the TEHG components. (a) The TEHG. (b-e) The rotor, stator, and wind cup of the S-TENG. (f-g) The magnet and coil of the EMG. (h) The F-TENG.

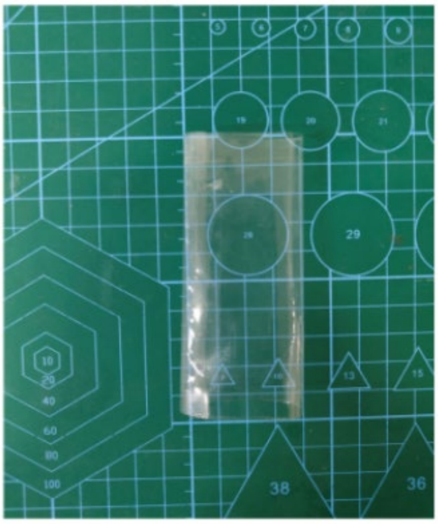

Figure S2. Arched FEP film.

Note S1: COMSOL simulation process of S-TENG

The modeling steps for the S-TENG in COMSOL Multiphysics are outlined below. First, begin by selecting the 2D spatial dimension in the COMSOL Model Wizard and add the Electrostatics (es) physics interface. For the study type, choose a Stationary study, as this assumes that the triboelectric layers have already made full contact and the surface charge density is saturated.

Next, construct the geometry of the S-TENG structure and enclose it within a rectangular air domain. Then, define the material properties by setting the relative permittivity of the entire rectangular air region to that of the air. Assign the relative permittivity of the Cu electrode to 1, FEP to 2.1, and Nylon to 3.5. Based on the charge conservation equation, set the surface charge density of the FEP film to 1 $\mu C/m^2$ and that of the Cu electrode to 0.5 $\mu C/m^2$, with the electric potential at infinity set to zero.

Following this, select a relatively fine mesh to balance computational precision and time requirements. Finally, compute the model to obtain the simulation results as shown in Figure 1c. During the processes (i)–(iv), as the negative triboelectric layer film slides over the positive triboelectric layer material, the electric potential on both sides of the electrode alternately rises and falls. When an external circuit is connected between the electrodes, repeating this cyclic process causes the potential difference between the electrodes to drive electron movement, generating a current in the external circuit. The modeling steps for the F-TENG in COMSOL are similar to those for the S-TENG.

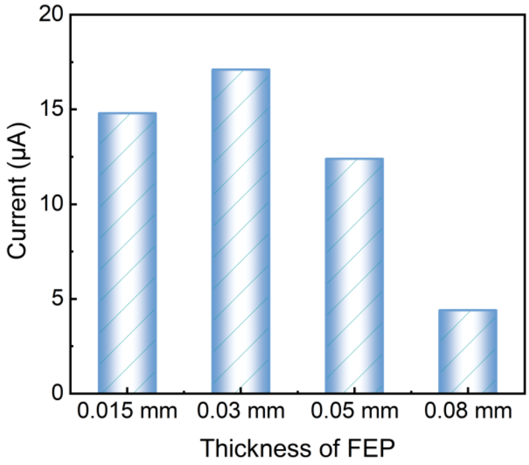

Figure S3. The I_{SC} of the S-TENG with different thicknesses of FEP under the wind speed of 6.0 $m \cdot s^{-1}$.

Table S1. Measured data of F-TENGs, including different channels, actual wind speed, frequency, calculated wind speed, and error.

Channel	Actual wind speed (m/s)	Frequency (Hz)	Calculated wind speed (m/s)	Error (%)
1	4.4	19.56	4.51	2.50%
1	5.4	28.26	5.50	1.85%
1	4.7	22.59	4.85	3.19%
1	5.4	27.72	5.43	0.56%
1	4.7	21.52	4.73	0.64%
1	6.7	40.66	6.90	2.99%
1	8.5	56.15	8.66	1.88%
2	8.5	54.5	8.47	0.35%
2	8.6	56.65	8.71	1.28%
2	9.0	58.53	8.93	0.78%
2	8.7	56.41	8.69	0.11%
2	8.0	52.37	8.23	2.88%
2	12.4	90.40	12.54	1.13%
3	5.6	29.36	5.62	0.36%
3	7.1	43.75	7.25	2.11%
3	8.6	57.40	8.80	2.33%
3	7.7	50.02	7.96	3.38%
3	8.1	50.35	8.00	1.23%
4	6.6	40.52	6.89	4.39%
4	5.5	36.46	5.43	1.27%
4	6.9	42.27	7.08	2.61%
4	6.4	37.47	6.54	2.19%
4	6.2	34.56	6.21	0.16%
5	5.9	31.69	5.88	0.34%
5	5.8	32.68	6.00	3.45%
5	5.7	29.70	5.66	0.70%
5	6.2	36.35	6.41	3.39%
5	7.8	50.35	8.00	2.56%
6	6.8	41.77	7.03	3.38%
6	7.9	50.46	8.01	1.39%
6	10.7	16.39	10.95	2.34%
6	9.1	61.69	9.29	2.09%
6	11.7	84.41	11.86	1.37%
6	10.6	76.33	10.95	3.30%
7	10.7	74.32	10.72	0.19%
7	10.4	70.43	10.28	1.15%
7	10.1	67.41	9.94	1.58%
7	7.5	44.56	7.34	2.13%
7	8.5	56.66	8.72	2.59%
7	7.8	49.52	7.91	1.41%
7	7.1	40.58	6.89	2.96%
8	7.4	46.22	7.53	1.76%
8	7.3	43.51	7.23	0.96%
8	7.6	48.42	7.78	2.37%
8	4.7	20.25	4.59	2.34%
8	4.9	24.59	5.08	3.67%
8	5.4	25.73	5.21	3.52%
8	7.8	49.88	7.95	1.92%
8	5.2	27.54	5.41	4.04%
